# How Seasonality May Shape Key Phenolic Compounds of *Eugenia punicifolia*: A Study Driven by NMR Metabolomics

**DOI:** 10.1021/acsomega.5c07927

**Published:** 2025-09-20

**Authors:** Kidney O G Neves, Samuel O Silva, Marinildo S Cruz, Francisco Célio M Chaves, Marcos B Machado, Alan Diego C Santos

**Affiliations:** † Laboratório de RMN, Central Analítica, 67892 Universidade Federal do Amazonas, 69067-005 Manaus, AM, Brazil; ‡ Embrapa Amazônia Ocidental, Empresa Brasileira de Pesquisa Agropecuária, 69010-970 Manaus, AM, Brazil; § Departamento de Química, Universidade Federal do Amazonas, 69067-005 Manaus, AM, Brazil; ∥ Grupo de Pesquisa em Química Sustentável e Metabolômica, 28118 Universidade Federal de Santa Maria, 97105-900 Santa Maria, RS, Brazil

## Abstract

*Eugenia punicifolia* DC., a medicinal plant rich in bioactive phenolics, has shown promising results regarding its potential in the prevention of type 2 diabetes. This study investigated the seasonal influence on the chemical profile of leaf extracts (methanol:ethanol:water, 3:1:1) using ^1^H NMR spectroscopy and multivariate analysis. Principal component analysis (PCA) and partial least-squares discriminant analysis (PLS-DA) revealed distinct metabolic patterns associated with rainy (Feb-May), dry (Sep-Nov), and transitional (Jan, Jun, Jul, and Dec) periods. The PLS-DA model showed high predictive power (*R* ^2^ > 0.85, *Q* ^2^ > 0.85), identifying quercetin, myricetin, gallic acid, catechin, and epigallocatechin as seasonal markers. Significant correlations were found between metabolite levels and environmental variables, such as temperature, rainfall, and sunlight exposure. These results demonstrate that abiotic factors regulate the biosynthesis of phenolic compounds, reflecting the plant’s adaptive responses. The study offers a scientific basis for optimizing harvest timing and enhances understanding of the ecological and pharmacological potential of *E. punicifolia*, providing valuable insights into the development of standardized herbal medicines and phytoproducts.

## Graphical abstract

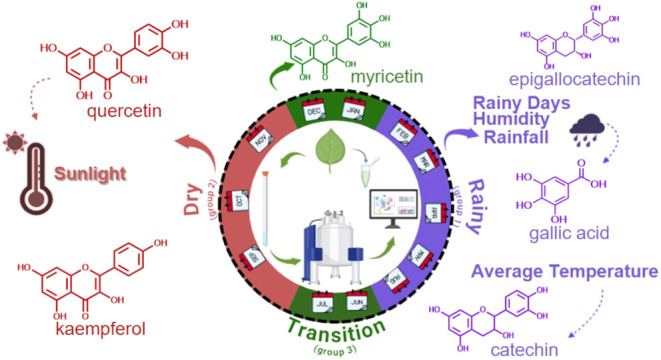

## Introduction

*Eugenia punicifolia* (Kunth) DC. is a species of Myrtaceae, native to and endemic in Brazil, with a broad distribution in the Amazon region. Its leaves are commonly commercialized and traditionally used in herbal medicine, especially for the treatment of diabetes mellitus type 2 (DM2). Known popularly as “vegetable insulin,” the species is part of a group of medicinal plants referred to as pedra-ume-caá.^1^ Studies investigating the scientific foundations of the traditional *E. punicifolia* usage have highlighted its antioxidant and antiglycation potential, attributing these pharmacological activities primarily to its flavonoid content.^2,3^

Flavonoids, widely distributed across the plant kingdom, represent the largest class of polyphenols shaped by long-term natural selection. These compounds exhibit a broad range of therapeutic effects in human—including antioxidant, anti-ischemic, anticancer, anti-inflammatory, and antibacterial activities—while also fulfilling essential physiological and ecological roles in plants.^4,5^ Flavonoids are synthesized in response to environmental pressures, with their biosynthesis modulated by both abiotic and biotic stressors.^6^ Consequently, even regular seasonal variations in climatic parameters (e.g., sunlight, temperature, precipitation) can influence the metabolic pathways involved in flavonoid production, ultimately altering the phytochemical composition of plants and, by extension, the pharmacological profile of herbal medicines.^7^ Correlating metabolite biosynthesis and accumulation with environmental conditions, a central concern of plant phenology, is inherently complex, as these responses are often dynamic, context-dependent, and multifactorial. Nonetheless, a deeper understanding of such patterns can provide valuable insights into ecosystem structure, function, and the services they offer.^8^

The influence of seasonal climatic variations on flavonoid content has been documented in several plants, including green tea (*Camellia sinensis*), Mediterranean species such as *Calamintha nepeta* L., *Helichrysum italicum* G., *Phillyrea latifolia* L., *Cistus incanus* L., and *Thymus longicaulis* C., Chinese prickly ash (*Zanthoxylum bungeanum* Maxim.), and grapes (*Vitis vinifera*).^4,9–14^ Such studies are essential not only for identifying the periods when flavonoid concentrations peak, thus optimizing harvest times to ensure maximum therapeutic efficacy, but also for elucidating the specific climatic parameters that modulate production. These insights can inform cultivation strategies and management practices to enhance the quality and consistency of medicinal plant materials.^7,9,10,12–14^

Building on this evidence, recent metabolomic investigations have reinforced the importance of seasonality and environmental factors in shaping the phytochemical composition of medicinal plants. Zanatta et al. conducted an integrated LC-MS and NMR metabolomic study of *Terminalia catappa* L., revealing seasonal variations in tannins, flavonoids, and triterpenes.^15^ Similarly, Pu et al. showed that both seasonal and interannual fluctuations markedly affect the accumulation of bioactive metabolites in the rhizomes of *Polygonatum cyrtonema* Hua, with direct implications for their medicinal quality.^16^ Crescenzi et al. highlighted how different fennel cultivars exhibit distinct metabolic patterns across seasons, while Nemadodzi et al. reported that growth conditions (open-field vs greenhouse) modulate the metabolome of *Solanum nigrum*.^17,18^ Collectively, these studies illustrate the growing use of metabolomics as a robust strategy to link phytochemistry, ecology, and pharmacology, providing a contemporary framework to contextualize seasonal responses in medicinal plants.

From a chemical perspective, monitoring phenolic compounds in complex matrices such as herbal medicines remains a challenging task. Nevertheless, NMR-based metabolomics has emerged as a powerful tool, offering a reliable snapshot of the downstream physiological state of biological systems.^19^ This approach has been successfully employed to track fluctuation in chemical composition influenced by environmental stressors.^20,21^

Our research group has applied NMR-based metabolomics to track seasonal modulations in *E. punicifolia* leaves. In an initial pilot study, multivariate analysis readily distinguished the chemical profiles of leaves collected during the dry and rainy seasons; however, the separation was primarily attributed to variations in primary metabolites.^22^ To more thoroughly assess the influence of seasonality on secondary metabolites, a follow-up study was conducted to identify an effective extraction solvent capable of capturing phenolic compounds. This allowed for a more accurate correlation between quantitative phenolic profiles and the pharmacological potential—specifically antioxidant, antiglycation, and antiviral activities—of *E. punicifolia* leaves collected across different Amazonia seasons.^2^

Therefore, this study aims to investigate how seasonal climatic variations throughout the year modulate the chemical profiles of *E. punicifolia* leaf extracts, using ^1^H NMR data integrated with multivariate statistical analysis. Unlike previous studies, leaf samples were collected monthly over the course of one year, allowing for a more detailed assessment of temporal fluctuations. The findings are expected to improve our understanding of how climatic parameters, such as sunlight exposure, temperature, precipitation, and relative humidity, influence the regulation of bioactive metabolites. Additionally, the study seeks to identify potential chemical markers associated with the plant’s adaptive defense mechanisms.

## Results and Discussion

### Chemical Profile of *E. punicifolia* Leaves Extract via NMR Spectroscopy

Aromatic compounds in *E. punicifolia* leaf extracts were identified through analysis of ^1^H NMR, ^1^H–^13^C HSQC, and ^1^H–^13^C HMBC spectra (Figure 1). The ^1^H NMR spectra revealed two singlets at δ_H_ 6.96 and δ_H_ 7.42, corresponding to gallic acid (**1**) and ellagic acid (**2**), respectively (Figure S1).^23^ Additionally, characteristic signals of flavonoids belonging to the flavanol (catechin and epigallocatechin) and flavonol (kaempferol, quercetin, and myricetin) classes were observed.^24^ Within the flavanol class, two distinct sets of signals were identified. The first set included δ_H_ 5.83 (d, 2.3 Hz), δ_H_ 5.93 (d, 2.3 Hz), δ_H_ 6.86 (d, 1.9 Hz), δ_H_ 6.87 (d, 8.1 Hz), and δ_H_ 6.74 (dd, 8.1 and 1.9 Hz,), corresponding to positions 6 and 8 of ring A, as well as 2′, 5′, and 6′ of ring B, respectively, in catechin (**3**).^25^ The second set included signals at δ_H_ 5.74 (d, 2.1 Hz), δ_H_ 5.89 (d, 2.1 Hz), and δ_H_ 6.40 (s), attributed to positions 6, 8, 2′, and 5′ of rings A and B, respectively, in epigallocatechin (**4**).^26^ For the flavonol class, signals from ring B were observed for kaempferol (**5**): H2′, H6′-δ_H_ 7.75 (d, 8.7 Hz); H3′, H5′-δ_H_ 6.91 (d, 8.7 Hz); quercetin (**6**): H1′-δ_H_ 7.30 (d, 2.1 Hz); H5′-δ_H_ 6.87 (d, 8.3 Hz); H6′-δ_H_ 7.25 (dd, 8.3 and 2.1 Hz); and myricetin (**7**): H2′, H6′-δ_H_ 7.01 (s).^25,27^ In addition, signals at δ_H_ 6.40 (d, 2.1 Hz) and δ_H_ 6.21 (d, 2.1 Hz), corresponding to the ring A hydrogens of flavonols, were also identified. The ^1^H–^13^C HMBC spectrum revealed long-range correlations between rings B and C, confirming the structures of the flavonoids identified by ^1^H NMR (Figures S1–S8).

**Figure 1:**
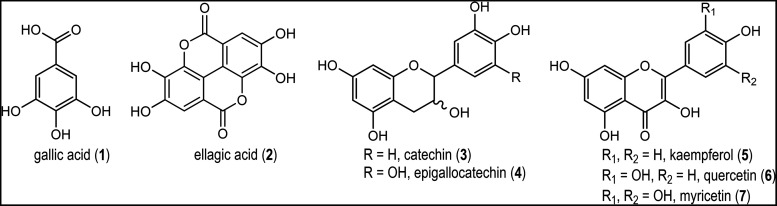
Compounds were identified by NMR analysis of MEW extracts from *E. punicifolia* leaves (500 MHz, DMSO-*d*_6_).

### Clustering Patterns by PCA

^1^H NMR spectra of extracts from *E. punicifolia* leaves collected over a 12-month period were analyzed by using PCA to identify clustering patterns and key discriminant compounds. According to the score plot shown in Figure 2, PC1 and PC2 together explained 38.21% of the total variance. The relatively low variance captured by the first two principal components indicates that the metabolic profiles are highly complex and are not dominated by a few variables. Nevertheless, PCA remained valuable for visualizing sample distribution and identifying meaningful trends, which were further supported and clarified by PLS-DA and heatmaps of significant metabolites. The analysis suggested a tendency toward the formation of three groups: **Group 1**, consisting primarily of samples collected in February, March, April, May, and August; **Group 2**, including samples from September, October, and November; and **Group 3**, comprising samples from January, June, July, and December.

**Figure 2:**
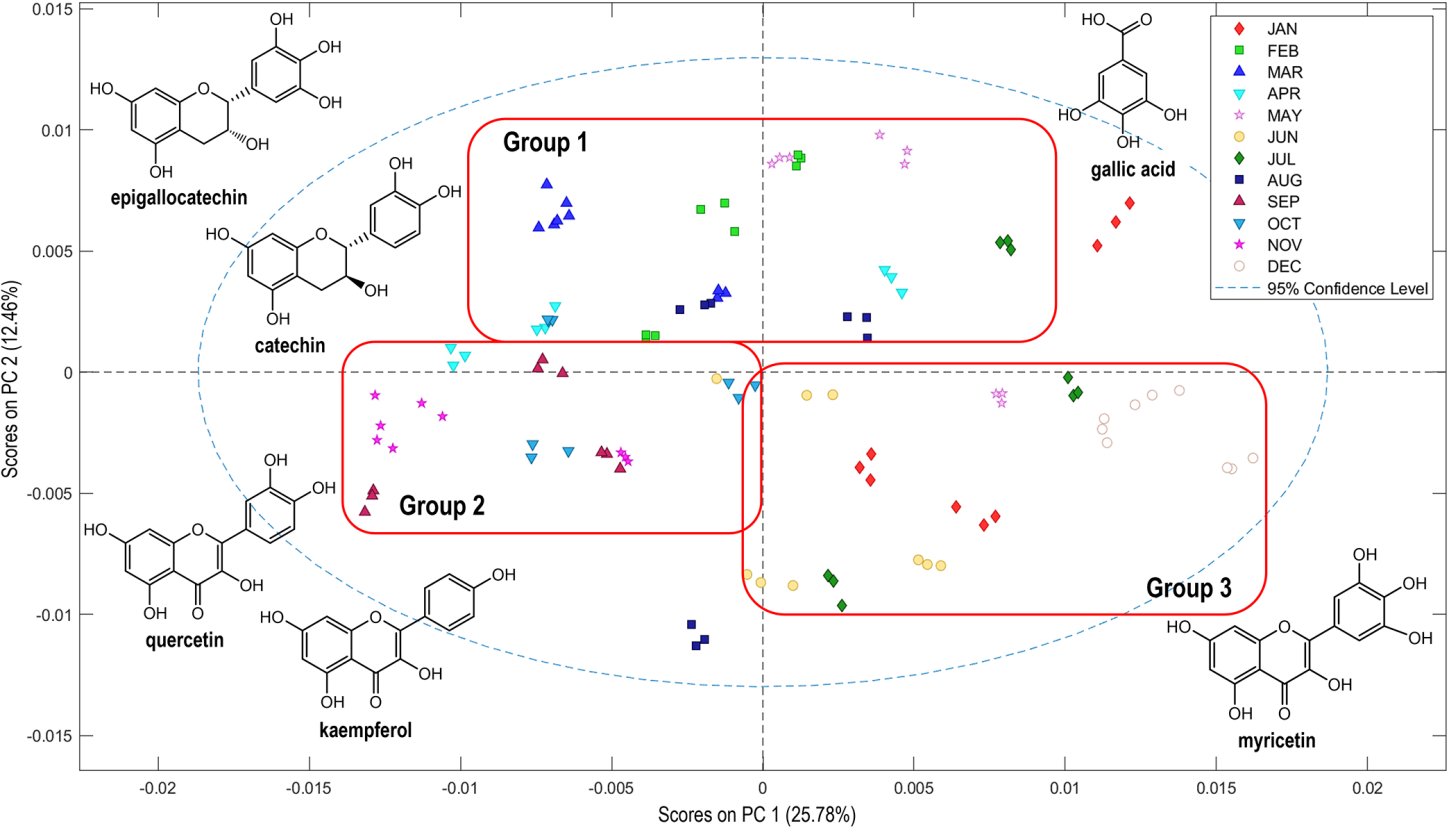
Principal component analysis (PCA) of *E. punicifolia* leaf extracts (MEW, 3:1:1). Score plot of PC1 (25.78%) versus PC2 (12.46%). The analysis of loading plots for PC1 and PC2, depicted in Figure S9, led to the assignment of key discriminant compounds, gallic acid, catechin, epigallocatechin, kaempferol, quercetin, and myricetin, for each group.

When analyzed alongside the meteorological data, the clustering of samples observed in the PCA suggested a pattern: the months comprising **Groups 1** and **2** exhibited an internal similarity in climatic parameters. Based on these similarities—and excluding August—**Group 1** (February, March, April, and May) was associated with the rainy season typical of the Amazonia winter, characterized by average temperatures below 28 °C, relative humidity above 85%, total precipitation exceeding 180 mm, more than 18 rainy days, and sunshine duration below 123 h (Table S1). In contrast, **Group 2** (September, October, and November) was linked to the dry season, corresponding to the Amazonian summer, with average temperatures above 28 °C, relative humidity below 80%, total precipitation under 115 mm, fewer than 10 rainy days, and sunshine duration above 134 h (Table S1). Similar approaches have been used in previous studies to distinguish between rainy and dry periods.^8,28,29^ Conversely, **Group 3** (June, July, January, and December) did not display a consistent climatic pattern. For instance, in terms of solar radiation, June and July were more similar to the dry periods, while January and December resembled the conditions observed in the rainy period. This variability prevents **Group 3** from being clearly categorized as either dry or rainy, making it more appropriately related to the transitional periods between the Amazonian summer and winter. This intermediate phase is marked by significant fluctuations in climatic parameters, with some months exhibiting characteristics of the rainy season and others resembling the dry season.

Regarding the chemical profile, analysis of the loading plot enabled the identification of associations between specific compounds and the clustering patterns observed (Figure S9). Gallic acid (δ_H_ 6.96, s), catechin (δ_H_ 5.93, d), and epigallocatechin (δ_H_ 5.89, d) were linked to samples in **Group 1**, while quercetin (δ_H_ 7.30, d) and kaempferol (δ_H_ 7.75, d) were associated with **Group 2**, and myricetin (δ_H_ 7.01, s) was linked to **Group 3** (Figure 2). When comparing samples from the rainy (**Group 1**) and dry (**Group 2**) periods, the analysis suggested that climatic parameters may significantly influence the biosynthesis of flavonoids, particularly flavanols and flavonols—two classes known for their strong association with the bioactivity of *E. punicifolia* leaves.^2^

A growing body of research has examined how environmental factors shape the biosynthesis of flavonoid in plants. Studies across diverse botanical models and analytical techniques have consistently supported this relationship. For instance, investigations in *V. vinifera* cultivars have shown substantially higher anthocyanin levels during winter than in summer.^13^ Similarly, in Chinese prickly ash bark, moderate yet significant Pearson correlations have been reported between flavonoid content and climatic variables such as average temperature (°C) and annual precipitation (mm).^4^ In *Tetrastigma hemsleyanum*, year-round monitoring of distinct flavonoids revealed that seasonal fluctuations influence biosynthetic selectivity.^7^ Collectively, these findings underscore the complex interplay between environmental factors and flavonoid profiles, supporting the associations observed in the present study.

### Classification by PLS-DA

To gain a more detailed understanding of the influence of climatic parameters on the clustering patterns observed in PCA, the ^1^H NMR spectra of samples from **Groups 1**, **2**, and **3** were subjected to PLS-DA analysis. In addition to validating the PCA results, PLS-DA provides loading plots and variable importance in projection (VIP) scores, which, when analyzed together, facilitate the identification of the chemical markers responsible for the observed groupings.

The analysis of the PLS-DA score plot revealed that the first latent variable predominantly contributed to classifying the samples in a pattern generally consistent with the clusters observed in the PCA, although it was not entirely overlapping. The quality of the PLS-DA model was assessed through the root-mean-square error of calibration (RMSEC), root-mean-square error of cross-validation (RMSECV), *Q* ^2^, and *R* ^2^. These diagnostics provide a statistically meaningful indicators of the model’s ability to discriminate between two classes of groups.^30,31^ Among these, RMSEC reflects how well the model fits the calibration data, while RMSECV assesses its performance on new data, thereby verifying its robustness.^32^ *R* ^2^ and *Q* ^2^ represent the explanatory and predictive capabilities in the model, respectively (Table 1).^31,33,34^

**Table 1:** Statistical Parameters Obtained from Cross-Validation on the PLS-DA Model, Including RMSECV, RMSEC, *R* ^2^, and *Q* ^2^ Values for the First Latent Variable

model	LV	X VC (%)	Y VC (%)	RMSECV	RMSEC	*Q* ^2^	*R* ^2^
A	1	23.43	87.86	0.20	0.17	0.84	0.88
B	1	26.55	90.30	0.18	0.15	0.88	0.90
C	1	30.38	90.41	0.18	0.15	0.87	0.90

Models A, B, and C, when evaluated based on the first latent variable, exhibited low RMSEC and RMSECV values (≤0.20), with minimal differences between calibration and cross-validation errors. These results indicate not only a good fit to the calibration set but also a satisfactory generalization performance for new data. An *R* ^2^ value exceeding 0.85 indicates that the models capture a substantial portion of the total variance of the calibration data, reflecting their robustness in describing the intrinsic variability of the system. Likewise, the high *Q* ^2^ values (>0.85) point to strong predictive capacity, consistent with the *R* ^2^ outcomes.^33,35,36^ The small gap between *R* ^2^ and *Q* ^2^ further reinforces the absence of overfitting, indicating that the discriminative and predictive performances remain reliable across the defined classes. To confirm these observations, RMSEC, RMSECV, *R* ^2^, and *Q* ^2^ were also analyzed for additional latent variables, which continued to demonstrate model stability and lack of overfitting, as illustrated in Figure 4.^36,37^

After model validation, variable importance projection (VIP) scores were used to evaluate the contribution of each compound to the chemical variation observed across seasons (Figures S13–S15). For interpretation of the VIP plots, only signals (δ_H_) with values greater than 1 were considered statistically significant.^35^ Based on the combined analysis of the loading graph and VIP scores, the compounds identified as significant contributors to the model were quercetin (δ_H_ 7.30, d), myricetin (δ_H_ 7.01, s), gallic acid (δ_H_ 6.96, s), catechin (δ_H_ 5.93, d), and epigallocatechin (δ_H_ 5.89, d).

The metabolites responsible for the patterns observed in models A-C are highlighted in Figure 3. Flavonoids from the flavanol (catechin and epigallocatechin) and flavonol (quercetin and myricetin) classes emerged as key discriminant compounds. In model A, catechin, epigallocatechin, and quercetin were responsible for the differentiation of samples from rainy (**Group 1**) and dry (**Group 2**) periods, consistent with PCA findings. In model B, catechin and epigallocatechin remained the primary markers of the rainy period, while myricetin was the principal compound distinguishing samples from the transition period (**Group 3**, January, December, June, and July). In model C, catechin and epigallocatechin played a central role in differentiating dry-period samples, whereas myricetin and gallic acid were prominent during the transitional period. These findings underscore that flavonoid biosynthesis in *E. punicifolia* leaves is modulated by seasonal variation.

**Figure 3:**
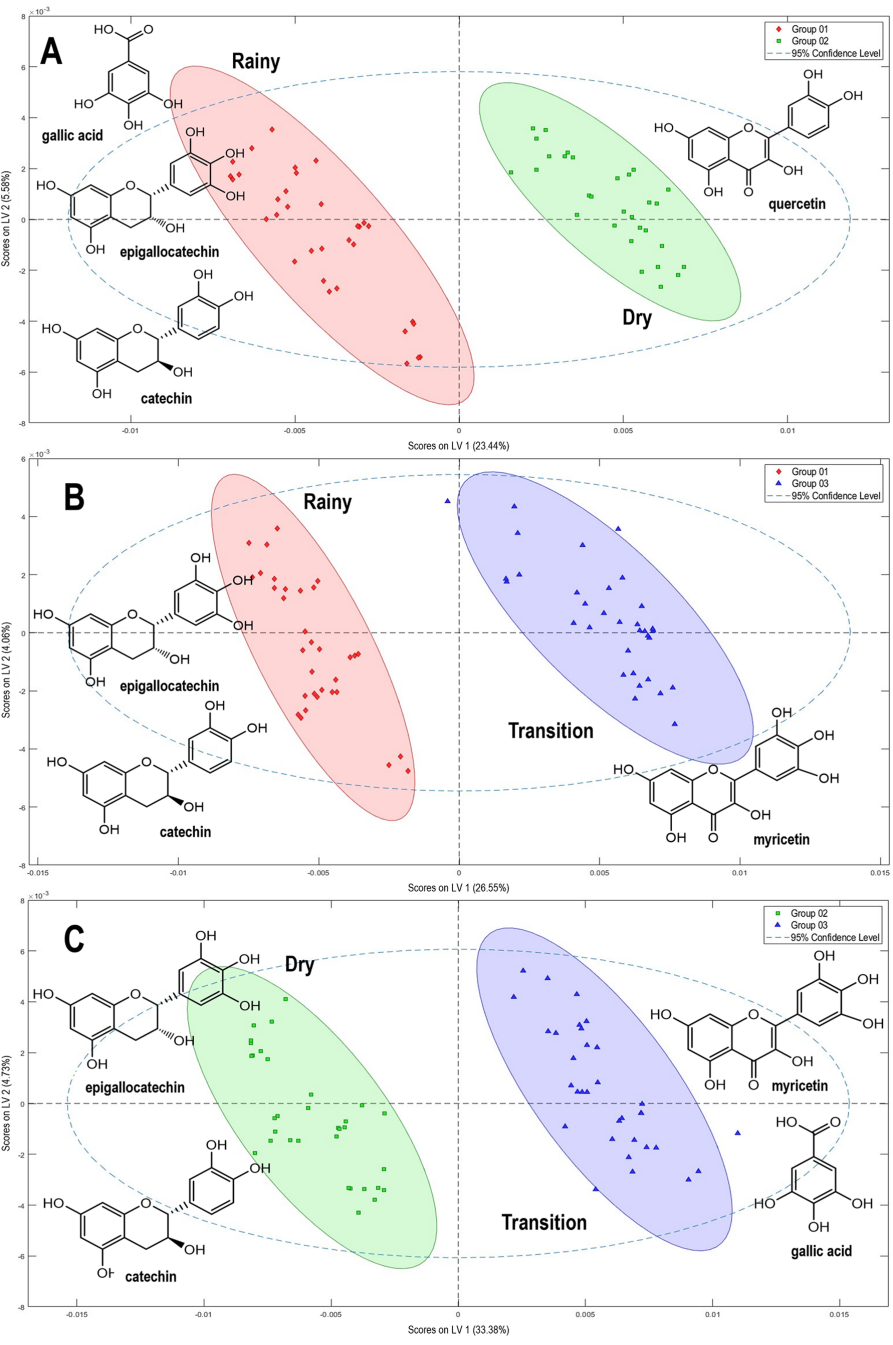
PLS-DA score plot illustrating the separation of samples based on the following models: **A**: **Group 1** vs **Group 2**, **B**: **Group 1** vs **Group 3**, and **C**: **Group 2** vs **Group 3**. The analysis of loading plots of LV1 and LV2, depicted in Figures S10–S12, led to the assignment of key discriminant compounds, gallic acid, catechin, epigallocatechin, quercetin, and myricetin.

### Correlation between NMR and Climate Data

The correlation between chemical composition and climatic parameters (Table S1) was assessed based on the principle that, in a properly calibrated ^1^H NMR spectrum, the signal area is proportional to the quantity of active nuclei present.^38,39^ The aromatic region of the spectrum was carefully aligned and segmented into buckets, with each representing a specific range of chemical shifts. This process is essential to minimize the spectral variability arising from differences in chemical composition and experimental acquisition conditions. By enhancing data consistency, the bucketing approach enables more reliable comparative analyses across samples (Figure 4).^40–42^

**Figure 4:**
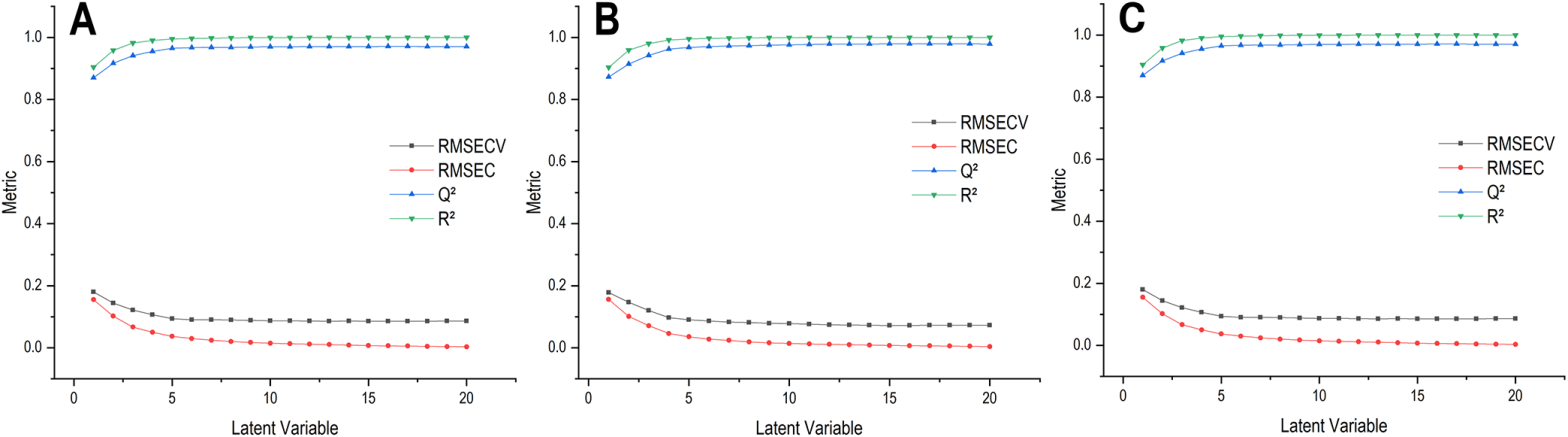
Evolution of RMSECV (black squares), RMSEC (red circles), *Q* ^2^ (blue triangles), and *R* ^2^ (green triangles) as a function of the number of latent variables used in the construction of the prediction models. Graphs were generated in OriginPro 2018 using data extracted from PLS-Toolbox Solo version 9.2 software. Metrics were derived from PLS-DA models comparing **Groups 1** vs **2** (**A**), **Groups 1** vs **3** (**B**), and **Groups 2** vs **3** (**C**).

The bucket areas corresponding to the signals of quercetin (δ_H_ 7.30, d), myricetin (δ_H_ 7.01, s), gallic acid (δ_H_ 6.96, s), catechin (δ_H_ 5.93, d), and epigallocatechin (δ_H_ 5.89, d) were normalized to the total area and used to evaluate variations across collection periods (Figure 5A). Furthermore, Pearson’s correlation coefficient was employed to quantify the strength of the correlations between the chemical profile and climatic parameters (Figure 5B).

**Figure 5:**
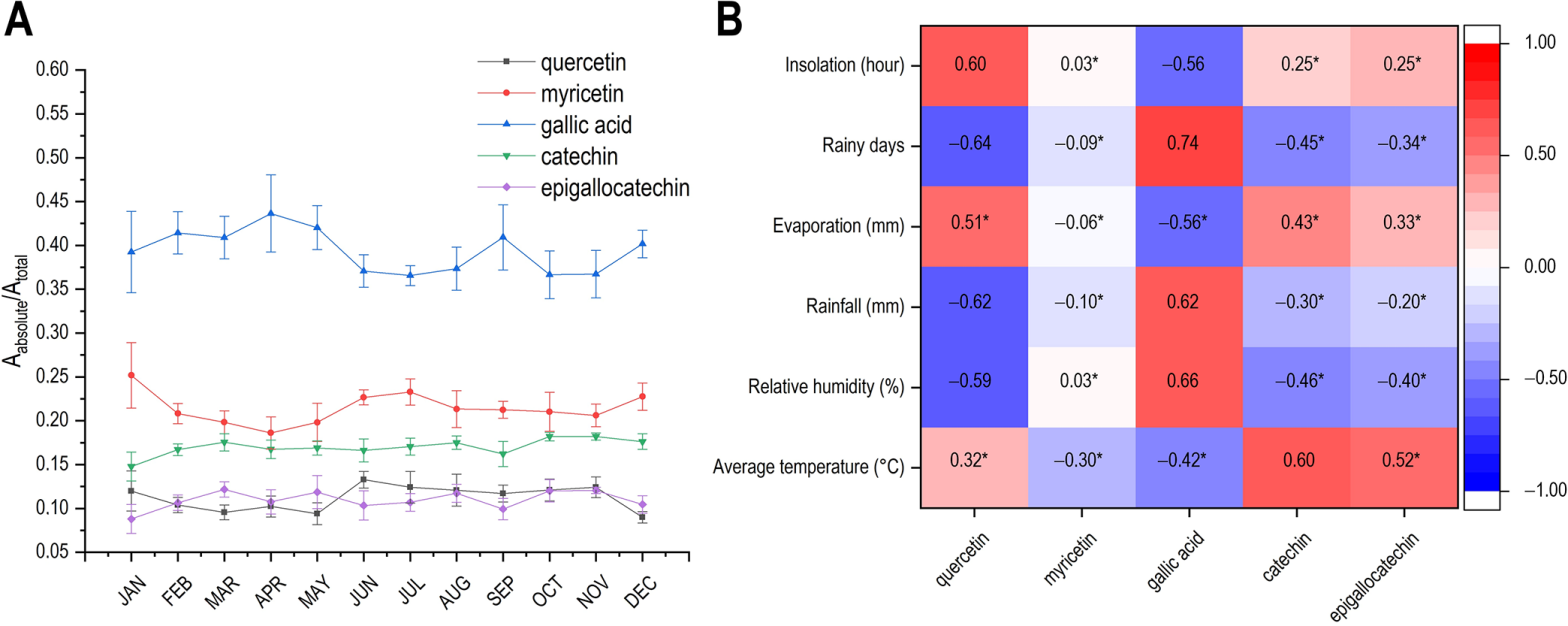
(A) Variation in the absolute area of the ^1^H NMR signal as a function of the collection month. (B) Heatmap of Pearson correlation coefficients between chemical composition and climatic parameters. *p-values >0.05 were considered not significant.

Figure 5A shows that the concentration of quercetin increases from June to November, suggesting an enhanced production during this period. In contrast, gallic acid displays higher and more stable concentrations between January and May, followed by a decline starting in June. Catechin and epigallocatechin exhibit similar seasonal patterns, with slightly higher relative concentrations from August to November. Therefore, except during the dry season, their levels tend to show a slight decline throughout the rest of the year. Also, myricetin reaches its highest levels during the transition months.

In Figure 5B, catechin production is positively correlated with rising temperatures (*r* = 0.60), while quercetin production appears to be favored by increased sunlight exposure (*r* = 0.60). In contrast, higher levels of relative humidity (*r* = −0.59), precipitation (*r* = −0.62), and number of rainy days (*r* = −0.64) are associated with reduced quercetin production in the leaves of *E. punicifolia*. Gallic acid production, on the other hand, exhibits the opposite trend, being stimulated under conditions of high relative humidity (*r* = 0.66), increased precipitation (*r* = 0.62), and a greater number of rainy days (*r* = 0.74). Conversely, periods of higher sunlight incidence are correlated with a reduction in gallic acid production (*r* = −0.56).

More pronounced levels of quercetin observed during part of the Amazonian summer months (September to November, Group 2) suggest a general biochemical strategy of *E. punicifolia* in response to increased sunlight irradiance and reduced water availability. This hypothesis is further supported by the moderate positive correlation (*r* = 0.60) between quercetin levels and sunlight exposure as well as by significant negative correlations with parameters associated with the rainy season (Figure 5B). In general, flavonoids contribute to the maintenance of reactive oxygen species (ROS) homeostasis.^44^ ROS are produced as part of the canonical plant response to environmental constraints such as UV radiation and drought stress.^43,44^ Acting through nonenzymatic antioxidant mechanisms, flavonoids help scavenge excess ROS, which—despite their role in promoting stomatal closure to limit water loss and light stress—can cause cellular damage, tissue death, and accelerated senescence when excessively accumulated.^43–45^ Among flavonoids, the role of quercetin in this context has been explored in various plant species.^44–46^

The association between gallic acid levels and the climatic conditions of the rainy season may be explained by the occurrence of both biotic and abiotic stresses. The regulatory role of gallic acid in enhancing abiotic stress tolerance in plants has been well documented, including its involvement in cold stress responses in soybean (*Glycine max*).^47–50^ In contrast, their role in mediating plant resistance against herbivores remains relatively underexplored. It is also important to consider that periods of high precipitation can directly increase the virulence of pathogens affecting aerial plant tissues, a phenomenon exacerbated by rainfall and elevated humidity levels.^51^ In this context, the production of gallic acid during the rainy season may offer significant advantages to *E. punicifolia*, as previous studies have suggested that gallic acid functions as an elicitor capable of triggering direct defense responses in plants by activating jasmonic acid signaling and the phenylpropanoid pathway.^52^ In addition to its regulatory functions, gallic acid also exhibits well-established antimicrobial and insecticidal properties.^53–55^

Catechin levels remained stable throughout the year, with a slight increase between September and November (Group 2), which may explain the observed correlation with higher average temperatures during the dry season. Assessing the influence of seasonality and temperature on catechins production appears to be a complex task, as discussed by Ahmed et al. 2019.^56^ Nevertheless, our findings align with trends reported in the literature, where elevated temperatures have been associated with increased catechin levels and the upregulation of genes involved in catechin biosynthesis in *C. sinensis* L.^57,58^ It is worth recalling that catechins contributed significantly to the grouping of samples associated with the rainy season, as revealed by a multivariate analysis. A significant increase in catechin content has been reported in *C. sinensis* during periods of intense rainfall, underscoring the influence of precipitation on flavonoid accumulation and its implications for tea production.^9^

Therefore, the statistical analysis of ^1^H NMR data, combined with climatic parameters, appears to be an effective strategy for assessing how the chemical composition of MEW extracts from *E. punicifolia* leaves is influenced by seasonality.

### Biosynthetic Considerations

We also aimed to examine how our findings align with the well-established biosynthetic pathways of quercetin and catechin, both of which share dihydroquercetin as a common precursor (Figure 6). The production of these compounds is dependent on the expression of specific enzymes. In the biosynthesis of quercetin, flavonol synthase (FLS) catalyzes the conversion of dihydroquercetin into quercetin. According to the literature, the expression of FLS is typically induced by high levels of UV radiation, which promotes the accumulation of flavonols—compounds that play key roles in protecting plants against UV damage and mitigating oxidative stress.^59,60^ In the catechin biosynthetic pathway, the enzymes dihydroflavonol 4-reductase (DFR) and leucoanthocyanidin reductase (LAR) are essential, with LAR specifically responsible for converting leucocyanidin into catechin (Figure 6). Increased LAR expression has been observed in *C. sinensis* under low light conditions.^61^

**Figure 6:**
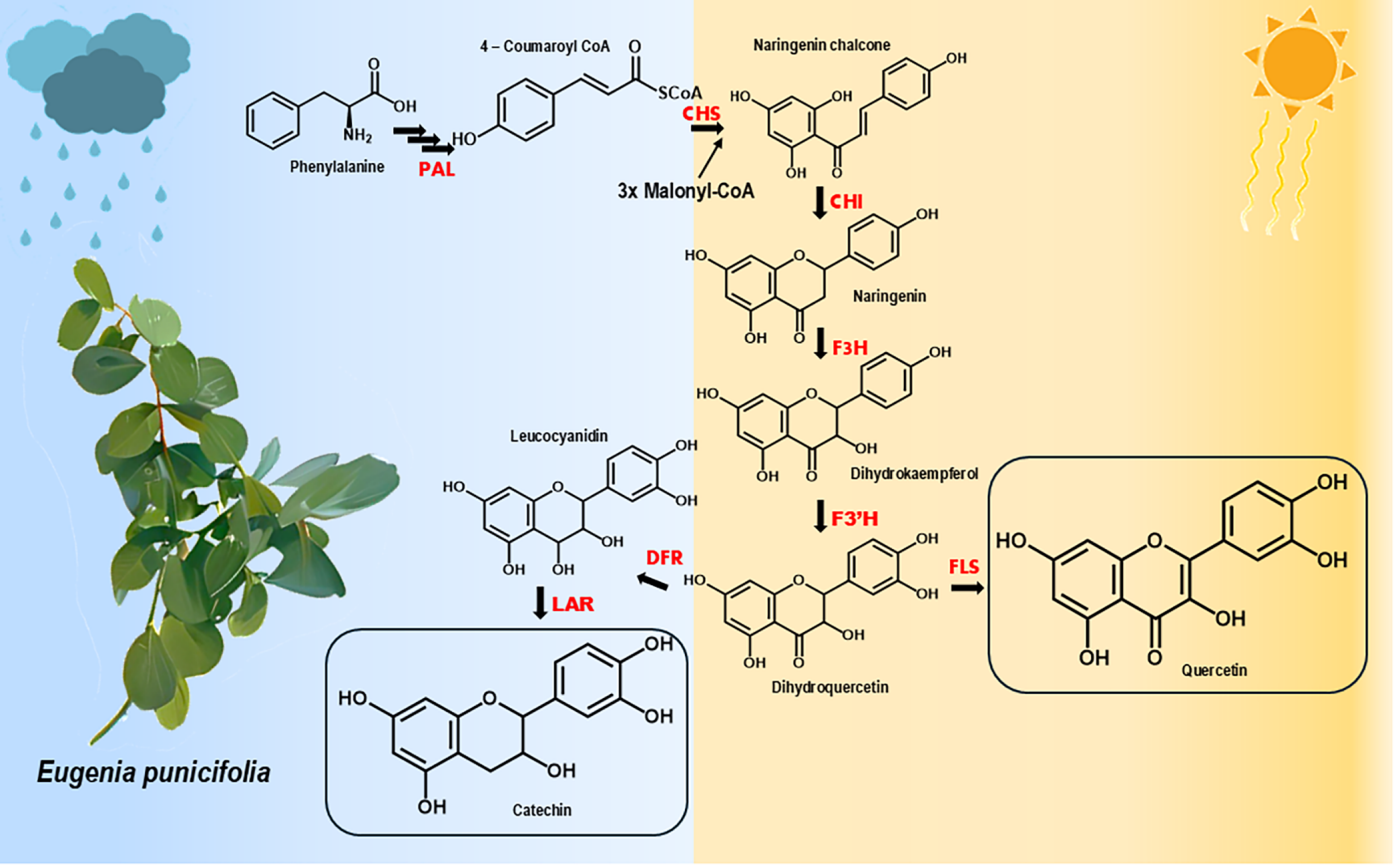
Schematic representation of the biosynthetic pathway for quercetin and catechin production, as influenced by seasonality. Enzyme abbreviations: ammonia-lyase (PAL), chalcone synthase (CHS), chalcone isomerase (CHI), flavanone 3-hydroxylase (F3H), flavonoid 3′-hydroxylase (F3′H), flavonol synthase (FLS), dihydroflavonol reductase (DFR), and leucoanthocyanidin reductase (LAR).

These biosynthetic insights support our findings regarding the seasonal distribution of quercetin and catechin, which were predominantly associated with dry and rainy periods, respectively. However, this is a complex issue, and further experimental evidence is required to fully elucidate the effect of seasonal variation. Future research should include multiyear sampling, experimental designs that isolate specific climatic variables, and transcriptomic analyses, among other approaches.

## Conclusions

The findings presented in this study highlighted the significant influence of seasonal climatic variations on the chemical profiles of *E. punicifolia* leaves. Quercetin, myricetin, gallic acid, catechin, and epigallocatechin were employed as chemical probes to assess how the species respond to environmental changes throughout the year. PCA revealed distinct sample groupings corresponding to the rainy, dry, and transitional periods of Amazon, which were further validated by PLS-DA model performance metrics. These results underscore the potential of phenolic compounds to reflect seasonal shifts in metabolite composition of *E. punicifolia*. Correlation analysis between climatic parameters and the chemical probe’s contents demonstrated that quercetin, gallic acid, and catechin were highly responsive to climatic variables such as average temperature, relative humidity, rainfall, number of rainy days, and sunlight exposure. Collectively, these findings provide a valuable scientific basis for understanding seasonal impacts on the chemical composition of medicinal plants, laying the groundwork for future studies of chemical ecology and spectrum-effect relationships of *E. punicifolia* and related species. Our results highlight the importance of optimizing the timing of plant material collection to enhance pharmacological potential. Furthermore, a logical and important next step for future research would be to explore the direct link between the observed seasonal chemical variations and their corresponding bioactivities through parallel bioassays.

## Methods

### Chemicals

Methanol (HPLC) and absolute ethanol (99.5% PA) used for plant material extraction were purchased from Sigma-Aldrich (St. Louis, MO, USA). Deuterated dimethyl sulfoxide (DMSO-*d*_6_, 99.9%) with tetramethylsilane (TMS, 0.05% v/v) for NMR analyses was obtained from Cambridge Isotope Laboratories, Inc. (Andover, Massachusetts, USA).

### Plant Material

Leaves of *E. punicifolia* were collected monthly throughout 2023 at the Brazilian Agricultural Research Corporation (Embrapa Amazônia Ocidental), located along Rodovia AM-010, Km 29 (2° 53′ 23″ S 59° 58′ 26″ W). Access to genetic heritage was registered (A82BD35) with the National System of Management of Genetic Heritage and Associated Traditional Knowledge (SisGen). From a plantation of 150 individuals, three trees were randomly selected each month. Fifteen leaves per tree were collected from the lower, middle, and upper canopy between 8:00 and 10:00 a.m. to ensure consistent and representative sampling. The plant material was air-dried at room temperature for 24 h and then macerated in liquid nitrogen, weighed, and stored at −80 °C until extraction.

### Environmental Data

Climate data for the 12-month study period were obtained from the Agroclimatology Laboratory of Embrapa Amazônia Ocidental and are provided in the Supporting Information (Table S1).

### Extraction Procedure

The extraction system was selected based on the methodology described by Neves et al. (2025).^2^ For each sample, 1.0 g was extracted in triplicate using 10 mL of a solvent system consisting of methanol (60%), ethanol (20%), and water (20%), hereafter termed the MEW system. Each extraction was performed four times, with sonication in an ultrasonic bath for 15 min, followed by centrifugation at 4000 rpm for 10 min (4226*g*). The resulting supernatant was collected and dried under a stream of nitrogen gas.

### Acquisition of NMR Spectroscopy Data

Twenty milligrams of *E. punicifolia* leaf extract was solubilized in 520 μL of deuterated dimethyl sulfoxide (DMSO-*d*_6_) and transferred to a 5 mm NMR tube. NMR analyses were performed on a Bruker Avance III HD NMR spectrometer (Bruker, MA, USA), operating at 11.7 T (500 MHz for ^1^H) and equipped with a 5 mm BBFO Plus SmartProbe with a *Z*-axis gradient. ^1^H NMR spectra were obtained at 25 °C by using the *zgpr* pulse sequence. The 90° pulse length was calculated individually for each sample. A total of 2 dummy scans and 32 scans were acquired with 32k data points, using a spectral width of 10 kHz, a relaxation delay of 15.0 s, and an acquisition time of 1.64 s. The residual water signal of DMSO-*d*_6_ (δ_H_ 3.36, s) was suppressed by using a power of 8.13 e^–5^ W, and the receiver gain was set to 64. Phase and baseline corrections were performed manually using TopSpin 3.6.3 software.^62^ The chemical shift values (ppm) of the ^1^H NMR spectra were referenced to the methyl signal of tetramethylsilane at δ_H_ = 0.0. ^1^H–^13^C correlations from HSQC and HMBC NMR experiments were acquired using coupling constants of 145 and 8 Hz for *J* (H,C—one-bond) and *J* (H,C—long-range), respectively.

### Multivariate Analysis

#### Principal Component Analysis

^1^H NMR spectra of the 36 leaf extract samples were acquired in triplicate, exported from TopSpin 3.6.3 software in.csv format, and imported into OriginPro 2018 software to build the data matrix.^62,63^ Chemometric analysis was carried out using the ^1^H NMR spectral region between 5.60 and 8.10 ppm, resulting in a matrix of 108 samples x 2048 variables. Principal component analysis (PCA) was performed using the PLS-Toolbox Solo 9.0 software.^64^ Spectra preprocessing included baseline correction using Automatic Whittaker Filter (asymmetry = 0.001, lambda = 100) and variable alignment with Correlation Optimized Warping (Slack 2, Segment Length 87). The data were normalized to the total spectral area and were mean-centered. These preprocessing methods were selected after some testing with reasonable methodologies. Score and loading plots were generated using the Singular Value Decomposition (SVD) algorithm.

#### Construction of the PLS-DA Calibration Model

To perform partial least-squares discriminant analysis (PLS-DA), the same data matrix used for PCA was employed. The samples were classified into three groups: **Group 1** (Rainy period)—February, March, April, and May; **Group 2** (Dry period)—August, September, October, and November; and **Group 3** (Transition period)—January, June, July, and December. In PLS-Toolbox Solo 9.0 software, the spectra were processed using baseline correction (Automatic Whittaker Filter with asymmetry = 0.001 and lambda = 100), variable alignment via Correlation Optimized Warping (Slack = 2, Segment Length = 87), and normalization to the total area followed by mean-centering.^64^ The processed data were then used to construct the PLS-DA calibration model. Cross-validation was performed by using the Venetian Blinds method with 10 splits and a blind thickness of 1. Statistical parameters, including the root-mean-square error of calibration (RMSEC), root-mean-square error of cross-validation (RMSECV), Q^2^, and R^2^, were analyzed using OriginPro 2018.^63^

### Pearson Correlation Coefficient Analysis

For this analysis, the ^1^H NMR spectra of the 108 *E. punicifolia* samples were initially exported to R-Studio software (version 2022.07.2).^65^ The spectral region from 5.60 to 8.10 ppm was aligned and divided into 0.03 ppm buckets with a 50% degree of freedom, resulting in a data table containing 108 samples and 101 variables. The bucket areas corresponding to the compounds quercetin (δ_H_ 7.30, d), myricetin (δ_H_ 7.01, s), gallic acid (δ_H_ 6.96, s), catechin (δ_H_ 5.93, d), and epigallocatechin (δ_H_ 5.89, d) were normalized to the total spectral area. Pearson correlation coefficients were calculated using Minitab 18.1 software, correlating the normalized bucket areas with climatic parameters: mean temperature (°C), relative humidity (%), precipitation (mm), evaporation (mm), number of rainy days, and sunshine duration (hours).^66^ Statistical significance was set at *p*-values <0.05. The resulting correlation coefficients were exported to OriginPro 2018 software and used to construct the heatmap.^63^

## Supplementary Material



## Notes

Climatic data collected monthly over the 12-month study period (Table S1); expanded aromatic region (5.70 to 8.00 ppm) of the ^1^H NMR spectrum of the MAE extract from *E. punicifolia* leaves (500 MHz, DMSO-*d*_6_) (Figure S1); ^1^H–^13^C HSQC spectrum of the MAE extract from *E. punicifolia* leaves (500 MHz, DMSO-*d*_6_) (Figure S2); expansion of the aromatic region of the ^1^H–^13^C HSQC spectrum of the MAE extract from *E. punicifolia* leaves (500 MHz, DMSO-*d*_6_) (Figure S3); expansion of the aromatic region of the ^1^H–^13^C HSQC spectrum of the MAE extract from *E. punicifolia* leaves (500 MHz, DMSO-*d*_6_) (Figure S4); ^1^H–^13^C HMBC spectrum of the MAE extract from *E. punicifolia* leaves (500 MHz, DMSO-*d*_6_) (Figure S5); expansion of the aromatic region of the ^1^H–^13^C HMBC spectrum of the MAE extract from Eugenia punicifolia leaves (Figure S6); expansion of the aromatic region of the ^1^H–^13^C HMBC spectrum of the MAE extract from Eugenia punicifolia leaves (Figure S7); main chemical shifts (ppm) observed in ^1^H NMR, ^1^H–^13^C HSQC, and ^1^H–^13^C HMBC spectra (500 MHz, DMSO-*d*_6_) (Figure S8); loadings plot of PC1 and PC2 discriminating the compounds responsible for the grouping of samples of *E. punicifolia* (500 MHz, DMSO-*d*_6_) (Figure S9); loadings plot of LV1 illustrating the compounds responsible for the classification of Groups 1 and 2 in the PLS-DA model (500 MHz, DMSO-*d*_6_) (Figure S10); loadings plot of LV1 illustrating the compounds responsible for the classification of Groups 1 and 3 in the PLS-DA model (500 MHz, DMSO-*d*_6_) (Figure S11); loadings plot of LV1 illustrates the compounds responsible for the classification of Groups 2 and 3 in the PLS-DA model (500 MHz, DMSO-*d*_6_) (Figure S12); VIP scores plot of classification of Groups 1 and 2 in the PLS-DA model (500 MHz, DMSO-*d*_6_) (Figure S13); VIP scores plot of classification of Groups 1 and 3 in the PLS-DA model (500 MHz, DMSO-*d*_6_) (Figure S14); and VIP scores plot of classification of Groups 2 and 3 in the PLS-DA model (500 MHz, DMSO-*d*_6_) (Figure S15) (PDF)
