# Supplementary material for: How Seasonality May Shape Key Phenolic Compounds of Eugenia punicifolia: A Study Driven by NMR Metabolomics
Source: ACS Omega. 2025 Sep 20;10(38):44738–48. doi: 10.1021/acsomega.5c07927 (PMC12489637; doi:10.1021/acsomega.5c07927)
Supplement: Supplementary file 1 [file ao5c07927_si_001.pdf]

Supplementary Information

How seasonality may shape key phenolic compounds of *Eugenia punicifolia*: a study driven by NMR-metabolomics

Kidney O. G. Neves [a], Samuel O. Silva [a], Marinildo S. Cruz [a], Francisco Célio M. Chaves [b], Marcos B. Machado [a,c], and Alan Diego C. Santos [d*]

[a]Laboratório de RMN, Central Analítica, Universidade Federal do Amazonas, 69067-005, Manaus, AM, Brazil

[b]Embrapa Amazônia Ocidental, Empresa Brasileira de Pesquisa Agropecuária, 69010-970, Manaus, AM, Brazil

[c]Departamento de Química, Universidade Federal do Amazonas, 69067-005, Manaus, AM, Brazil

[d]Grupo de Pesquisa em Química Sustentável e Metabolômica, Universidade Federal de Santa Maria, 97105-900, Santa Maria, RS, Brazil

Corresponding authors:

Alan Diego C. Santos: https://orcid.org/0000-0002-3877-0313

alan.santos@ufsm.br*

Table S1. Climatic data collected monthly over the 12-month study period. Air temperature and relative humidity were reported as monthly mean values, while precipitation, number of rainy days, evaporation, and insolation were expressed as the cumulative totals for each month. The climatic data were provided by the Brazilian Agricultural Research Corporation (Embrapa) Western Amazon.

Months	Temperature (°C)			Relative humidity (%)	Rainfall (mm)	Rainy days	Evaporation (mm)	Insolation (hour)
	**Maximum**	**Minimum**	**Average**					
January	30.7	22.4	25.6	88.8	345.1	23	47.5	70.9
February	31.2	22.5	26.1	88.8	460.0	22	40.7	64.8
March	31.8	23.0	26.6	87.6	384.3	20	54.5	92.9
April	32.9	23.2	27.0	88.6	297.1	25	53.1	97.7
May	33.6	23.6	27.7	86.5	183.4	19	56.8	123.2
June	32.4	22.7	26.8	86.4	101.6	16	55.9	140.9
July	34.1	22.8	27.8	79.6	61.9	6	93.6	208.9
August	35.3	23.2	28.6	77.1	85.1	8	112.0	212.2
September	36.1	23.3	28.9	79.1	46.4	8	124.5	171.9
October	36.3	23.8	29.3	74.3	67.5	7	140.2	143.7
November	35.0	23.9	28.8	79.4	114.8	7	105.2	134.5
December	31.8	23.2	27.0	89.2	268.6	21	54.0	67.1

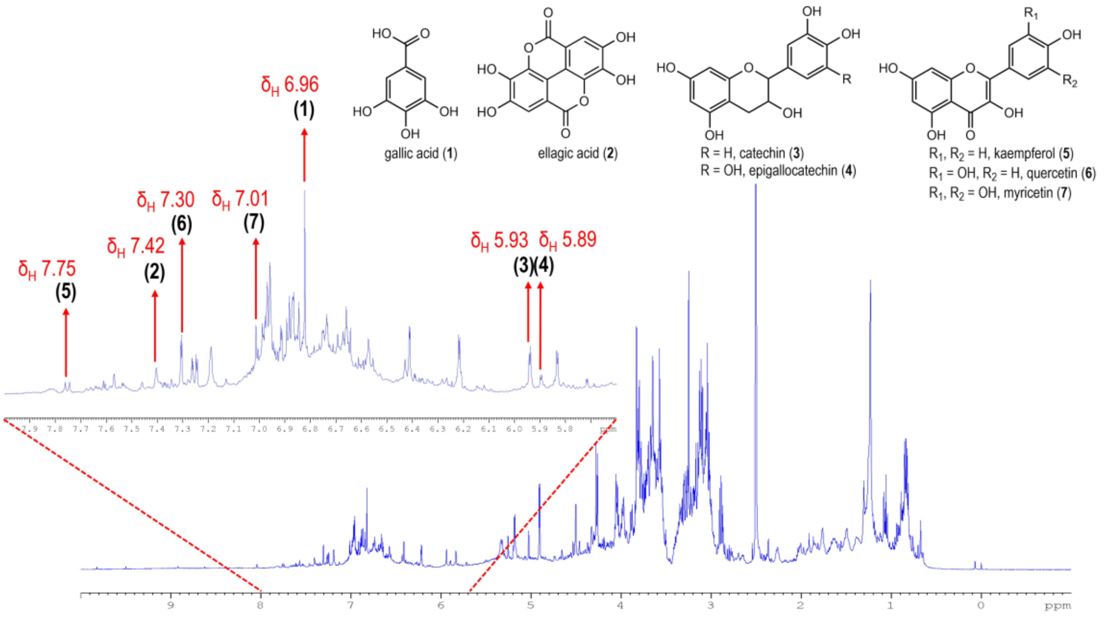

Figure S1. Expanded aromatic region (5.70 to 8.00 ppm) of the ^{1}H NMR spectrum of the MAE extract from *Eugenia punicifolia* leaves (500MHz, DMSO-d_6).

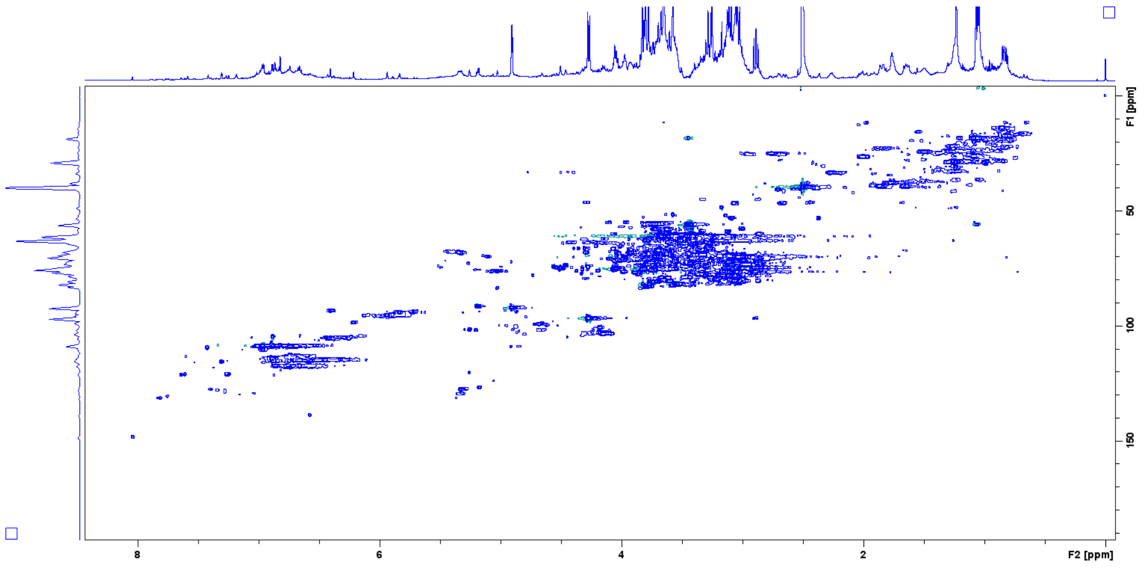

Figure S2. ^{1}H-^{13}C HSQC spectrum of the MAE extract from *Eugenia punicifolia* leaves (500MHz, DMSO-d_6).

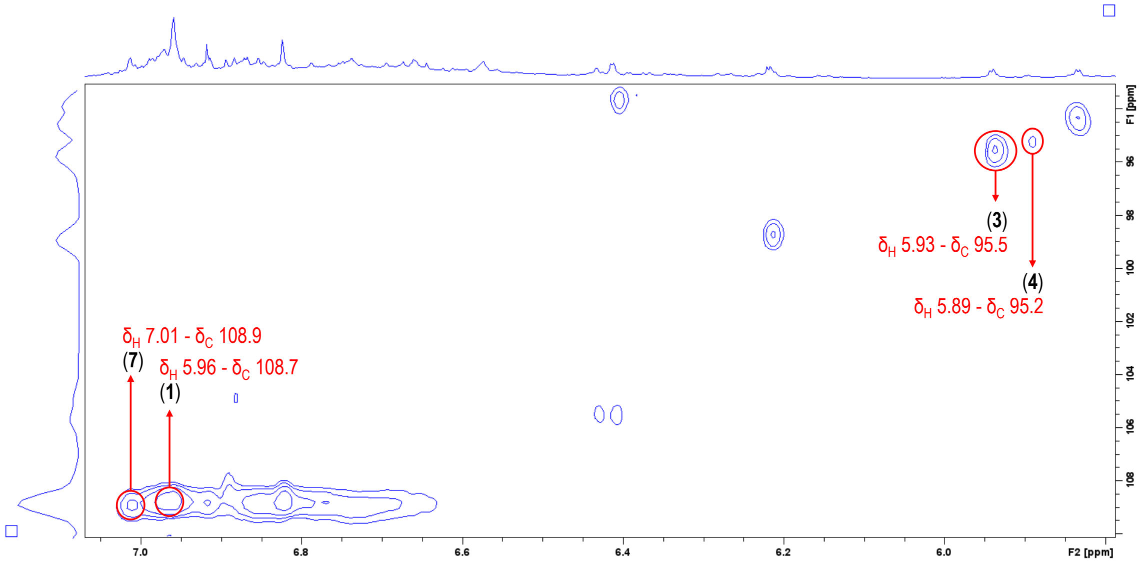

Figure S3. Expansion of the aromatic region of the ^{1}H-^{13}C HSQC spectrum of the MAE extract from *Eugenia punicifolia* leaves (500MHz, DMSO-d_6).

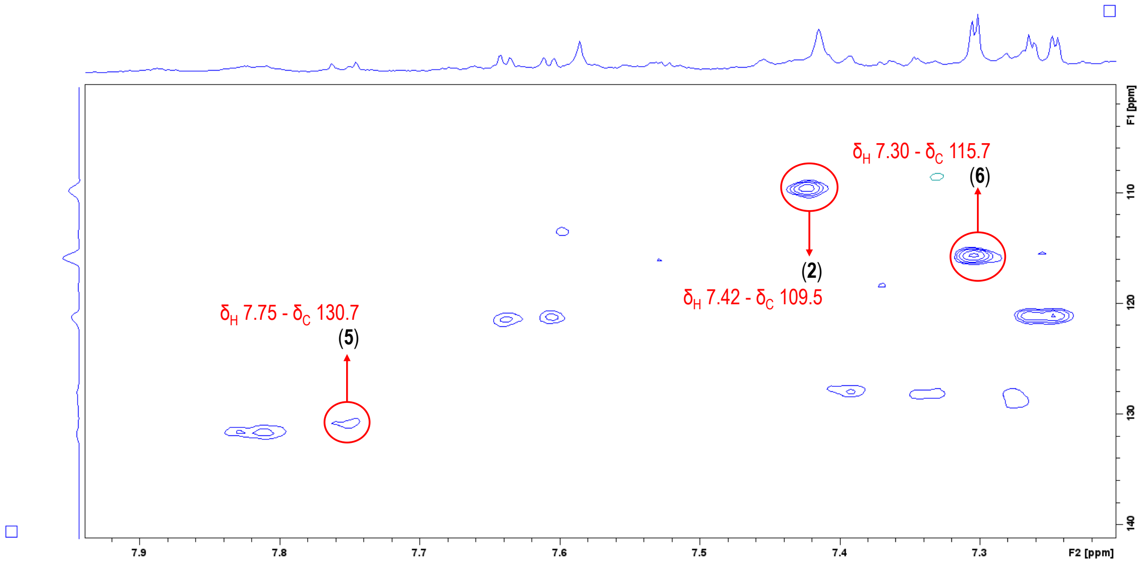

Figure S4. Expansion of the aromatic region of the ^{1}H-^{13}C HSQC spectrum of the MAE extract from *Eugenia punicifolia* leaves (500MHz, DMSO-d_6).

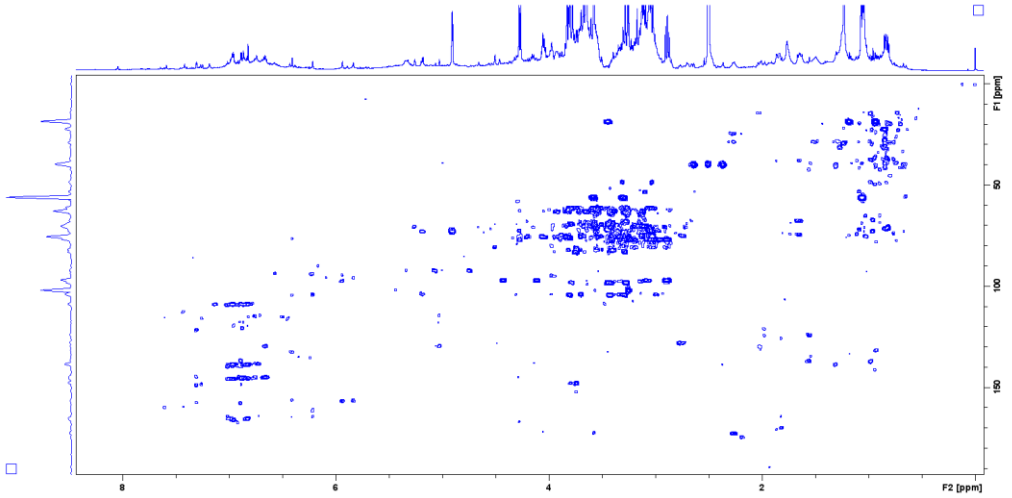

Figure S5. ^{1}H-^{13}C HMBC spectrum of the MAE extract from *Eugenia punicifolia* leaves (500MHz, DMSO-d_6).

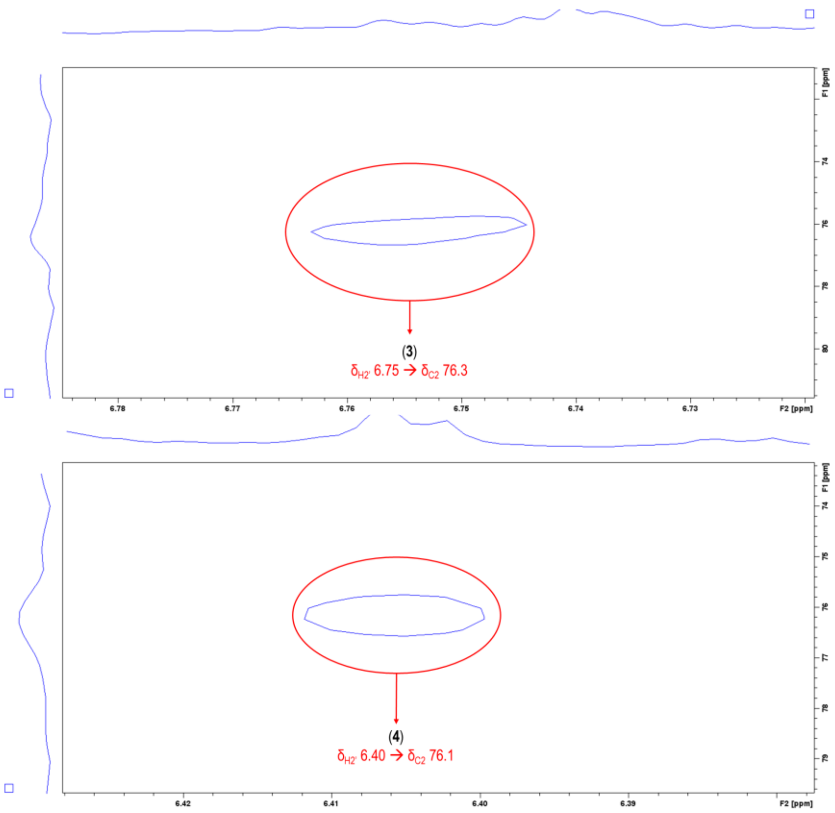

Figure S6. Expansion of the aromatic region of the ^{1}H-^{13}C HMBC spectrum of the MAE extract from *Eugenia punicifolia* leaves. Key correlation for confirmation of binding between catechin and epigallocatechin B and C rings (500MHz, DMSO-d_6).

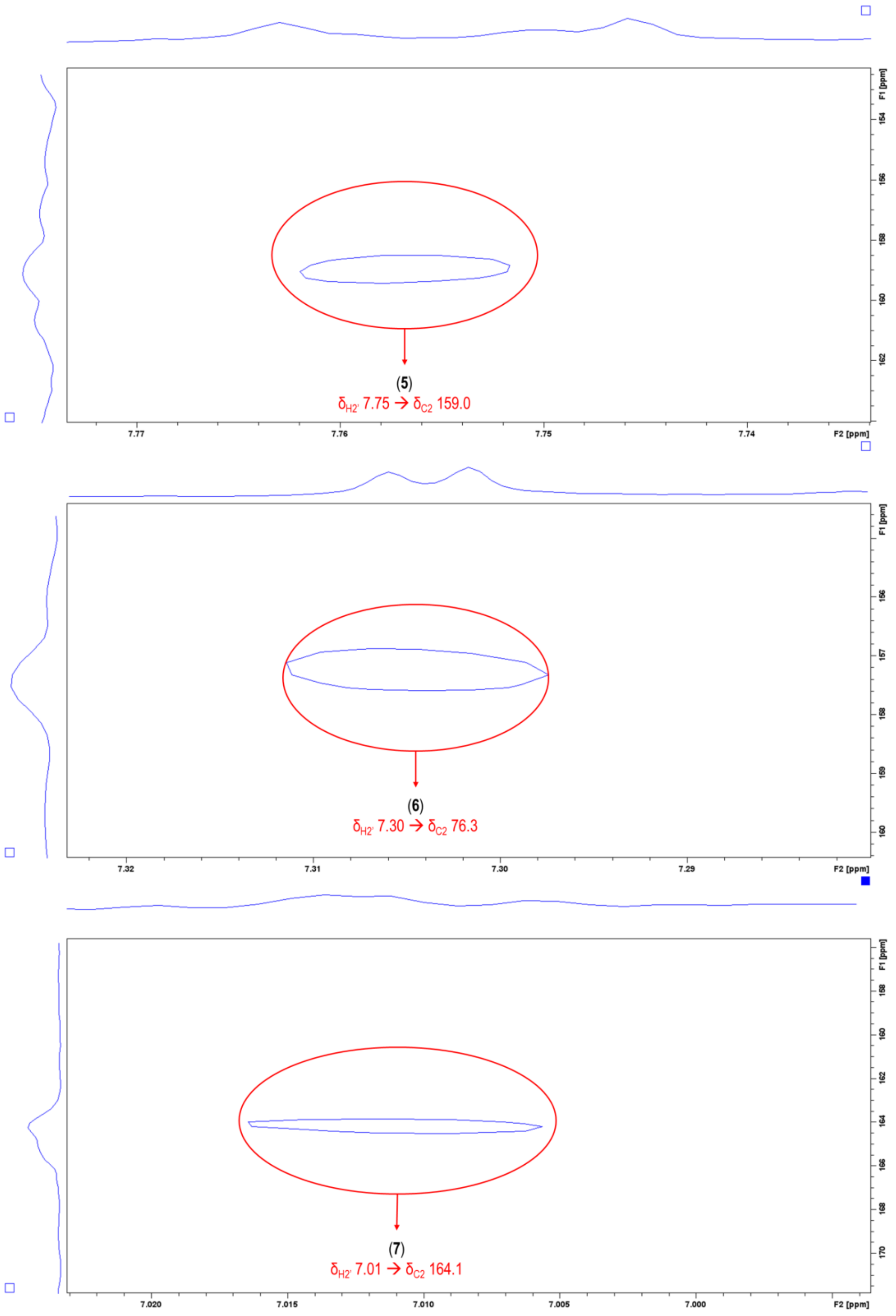

Figure S7. Expansion of the aromatic region of the ^{1}H-^{13}C HMBC spectrum of the MAE extract from *Eugenia punicifolia* leaves. Key correlation for confirmation of binding between apigen, myricetin and quercetin B and C rings (500MHz, DMSO-d_6).

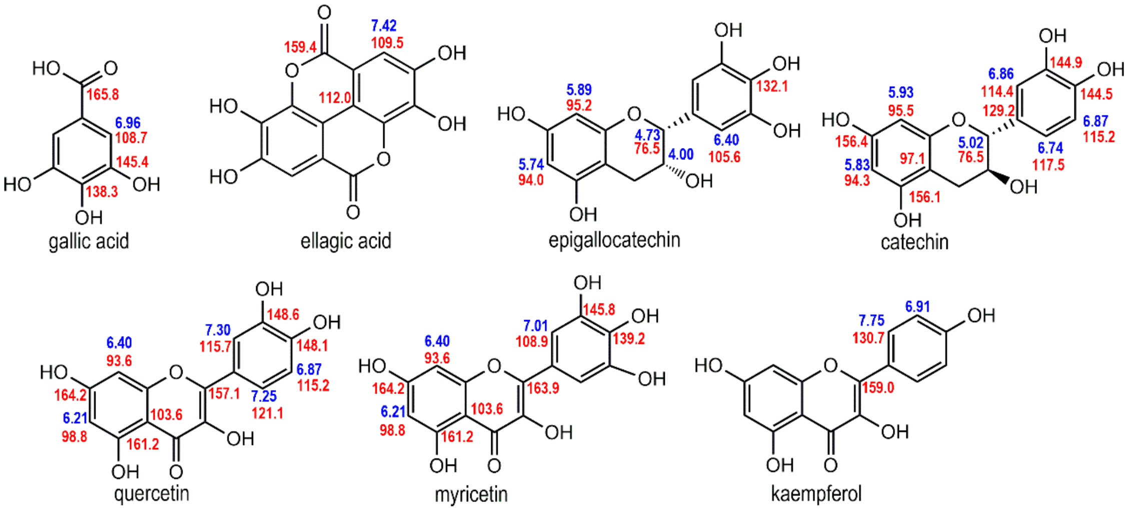

Figure S8. Main chemical shifts (ppm) observed in ^{1}H NMR, ^{1}H-^{13}C HSQC and ^{1}H-^{13}C HMBC spectra (500 MHz, DMSO-d_6).

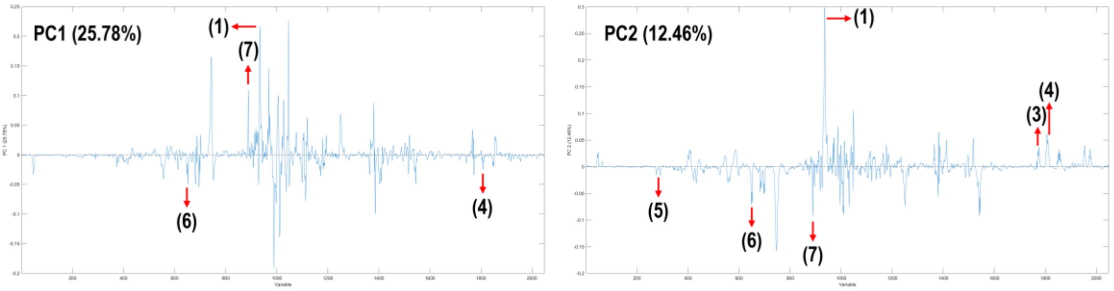

Figure S9. Loadings plot of PC1 and PC2 discriminating the compounds responsible for the grouping of samples of *E. punicifolia* (500MHz, DMSO-d_6).

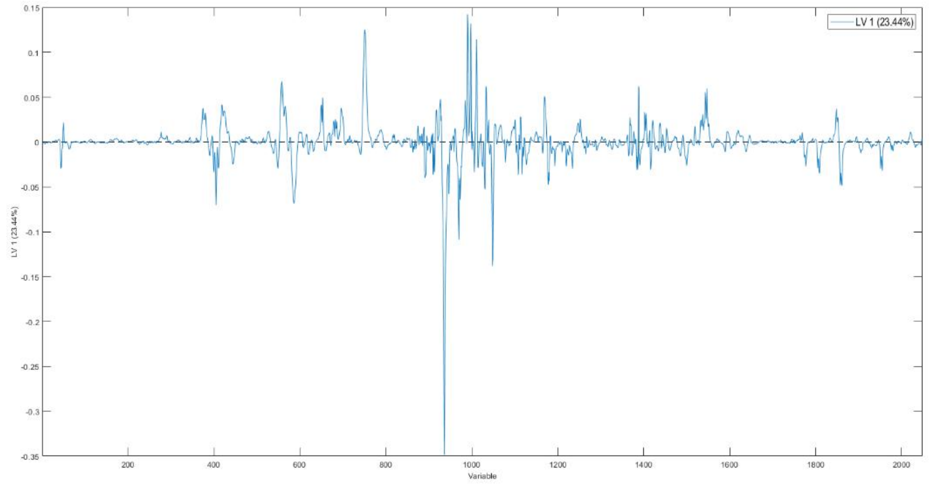

Figure S10. Loadings plot of LV1 illustrating the compounds responsible for the classification of **Groups 1** and **2** in the PLS-DA model (500MHz, DMSO-d_6).

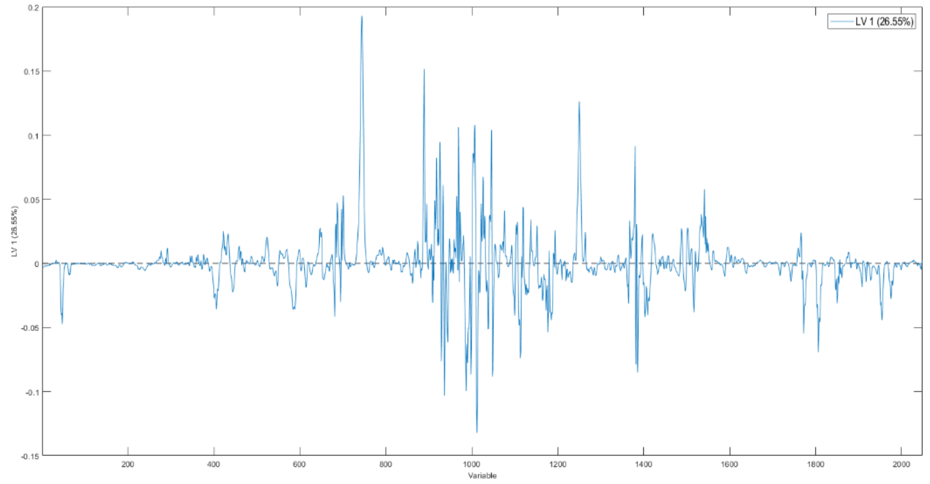

Figure S11. Loadings plot of LV1 illustrating the compounds responsible for the classification of **Groups 1** and **3** in the PLS-DA model (500MHz, DMSO-d_6).

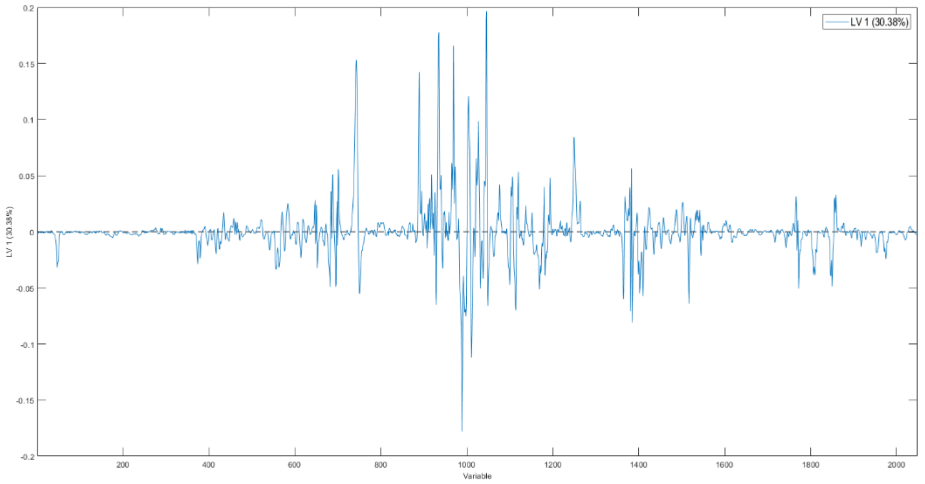

Figure S12. Loadings plot of LV1 illustrates the compounds responsible for the classification of **Groups 2** and **3** in the PLS-DA model (500MHz, DMSO-d_6).

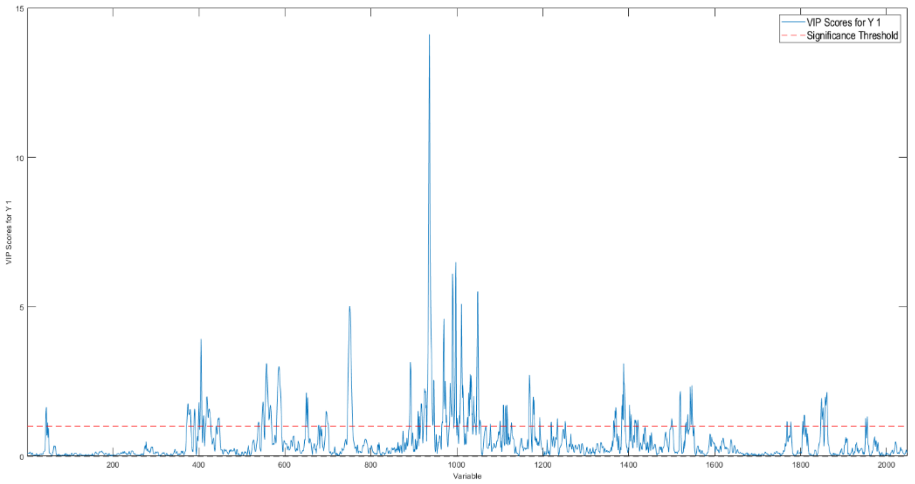

Figure S13. Vip scores plot of classification of **Groups 1** and **2** in the PLS-DA model (500MHz, DMSO-d_6).

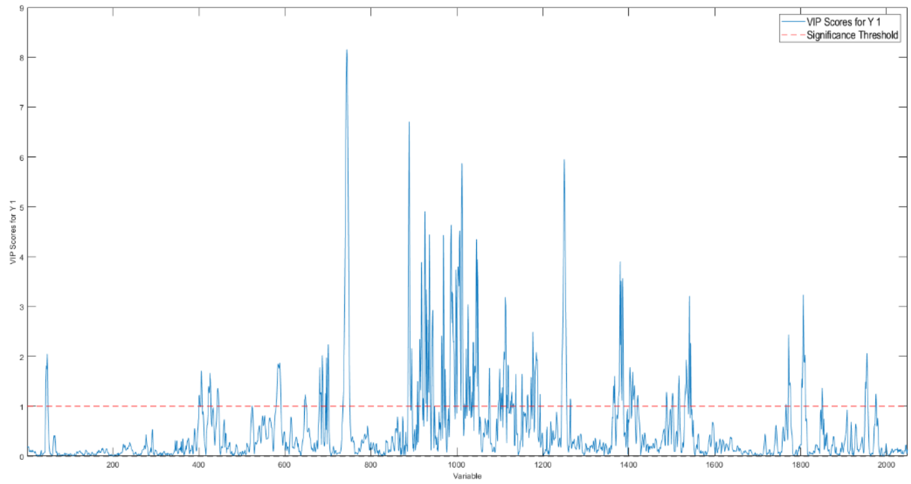

Figure S14. Vip scores plot of classification of **Groups 1** and **3** in the PLS-DA model (500MHz, DMSO-d_6).

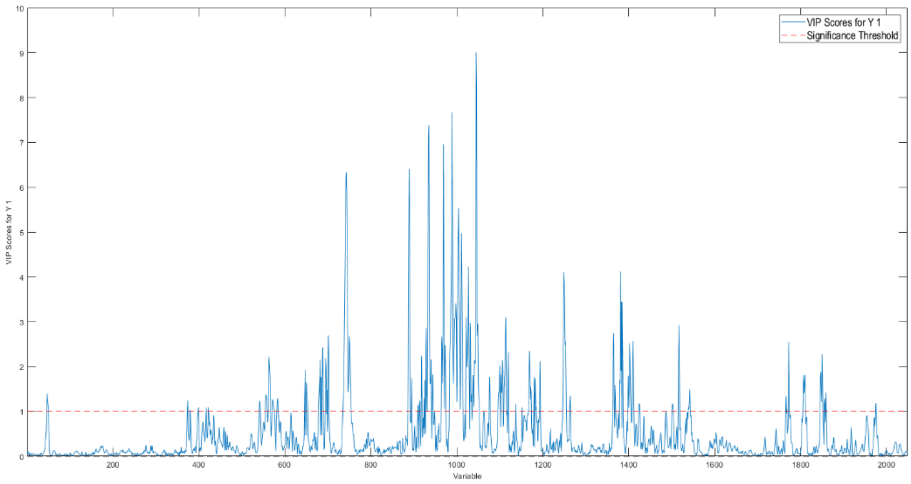

Figure S15. Vip scores plot of classification of **Groups 2** and **3** in the PLS-DA model (500MHz, DMSO-d_6).
